# Dr. Subhas Mukhopadhyay (1931-1981): A Pioneer in the Field of Reproductive Medicine

**DOI:** 10.7759/cureus.68608

**Published:** 2024-09-04

**Authors:** Anjani Mahesh Kumar Cherukuri, Gnaneswar Pendurthi, Chinmay Rao Chanda

**Affiliations:** 1 Internal Medicine, Guntur Medical College, Guntur, IND; 2 Community Medicine, Guntur Medical College, Guntur, IND

**Keywords:** assisted reproductive technology (art), test tube baby, embryo cryo-preservation, in vitro fertilization ivf, historical vignette

## Abstract

Dr. Subhas Mukhopadhyay achieved a significant milestone in reproductive medicine by creating India’s first test-tube baby, just 67 days after British researchers made a similar breakthrough. Despite this remarkable achievement, Dr. Mukhopadhyay faced substantial discredit, ridicule, and forced silence, which tragically led to his untimely death by suicide. It was only through the persistent efforts of Dr. T. C. Anand Kumar that Dr. Mukhopadhyay’s pioneering work was eventually recognized, and he was credited with this groundbreaking accomplishment in India.

## Introduction and background

Although most couples trying to conceive will achieve pregnancy as anticipated or even sooner, about 9% of couples of childbearing age experience unexpected infertility [1]. Assisted reproductive technology (ART) offers a remarkable solution for these couples, with in vitro fertilization (IVF) standing out as the most prevalent and effective form of ART [2]. On July 25, 1978, Louise Brown, the world’s first IVF baby, was born in the UK, thanks to the groundbreaking efforts of Dr. Robert G. Edwards and Dr. Patrick Steptoe [3].

Just days after the world’s first test-tube baby was born in the UK, an Indian team from Kolkata, led by the distinguished cryobiologist Dr. Subhas Mukherjee (Mukhopadhyay) and the prominent gynecologist Dr. Saroj Bhattacharya, reported the birth of “Durga” on October 3, 1978, through a comparable IVF procedure [4,5]. Despite this remarkable achievement, Dr. Mukherjee did not receive the same recognition as Dr. Edwards. Instead, his work faced skepticism, ridicule, and rejection from scientists and peers.

An expert committee established by the West Bengal government, which included a radiophysicist, a neurologist, a gynecologist, and a physiologist with no expertise in IVF, ultimately discredited Dr. Mukherjee’s research. As a result, he was barred from international scientific events and from sharing his findings. He was repeatedly transferred and prevented from continuing his research, which contributed to his health deterioration, including a heart attack. Overwhelmed by mental pressure, insults, and defamation, he took his own life on June 18, 1981. In his final note, he reportedly wrote, “I can’t wait every day for a heart attack to kill me” [2].

It was not until 1997 that Dr. T. C. Anand Kumar, former Director of the Institute of Research in Reproduction, Mumbai, reviewed Mukherjee’s papers and handwritten notes on his technique. Dr. Kumar, who had been instrumental in the birth of another test-tube baby in 1986, not only exonerated Dr. Mukherjee from accusations of fraud but also wrote extensively about his groundbreaking achievements.

## Review

Early life and scholastic foundations

Dr. Mukherjee was born on January 16, 1931, in Hazaribagh, Jharkhand, into a Brahmin family. His father, Dr. Satyendra Nath Mukherjee, was a distinguished radiologist, and his mother, Mrs. Jyotsna Devi, supported his early education. Dr. Mukherjee completed his matriculation with first-division honors from Bow Bazar High School in Calcutta and his Intermediate Science from Surendra Nath College. He pursued a BSc (Hons) in Physiology at Presidency College, Calcutta University, and earned his MBBS degree from Calcutta Medical College in 1955. At Calcutta Medical College, he excelled in obstetrics and gynecology, receiving the “Himangini” Scholarship and College Medal [2].

**Figure 1:**
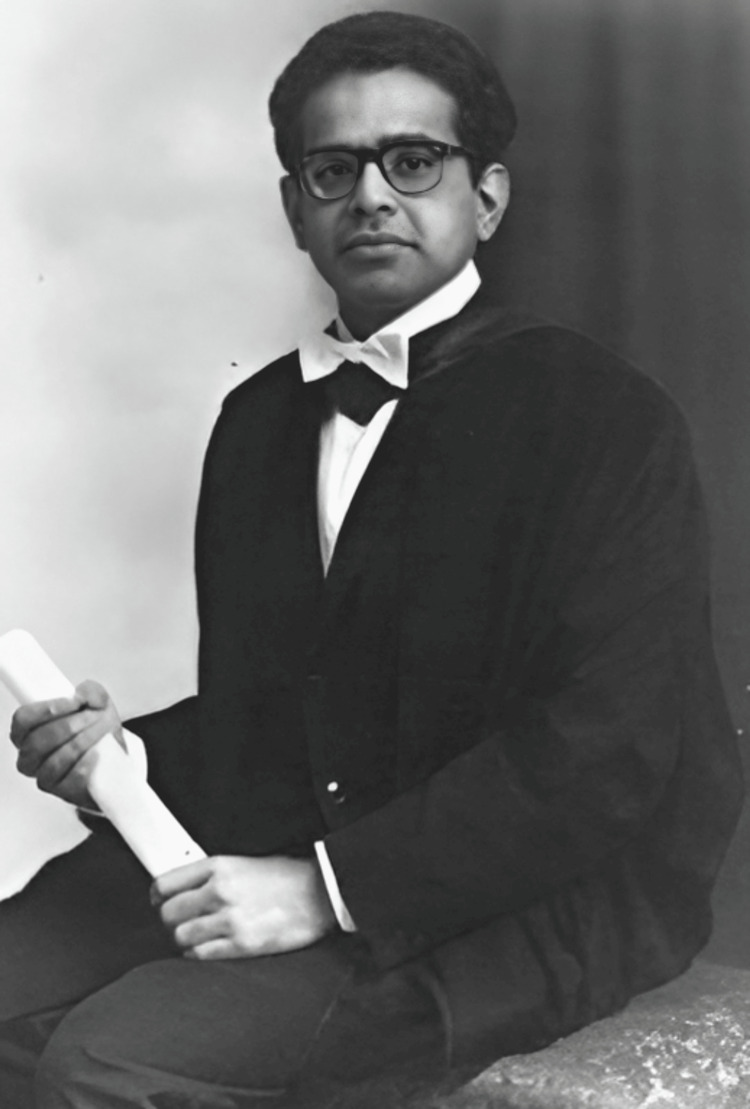
Dr. Subhas Mukhopadhyay Source: Wikipedia Author: Unknown Permission: Public domain

Dr. Mukherjee obtained his first PhD in reproductive physiology from Rajabazar Science College in 1958, under the mentorship of Prof. Sachidananda Banerjee (Figure 1). A decade later, he earned a second PhD in reproductive endocrinology from the University of Edinburgh, where he studied under the esteemed reproductive physiologist Prof. John A. Loraine.

Dr. Mukherjee’s pioneering innovations in IVF

On October 3, 1978, Dr. Mukherjee, along with Prof. Sunit Mukherjee, a cryobiologist, and Dr. Saroj Kanti Bhattacharya, a gynecologist, proudly announced the birth of the world’s second test-tube baby, Durga (Kanupriya Agarwal), in Calcutta. This achievement came just 67 days after R.G. Edwards and Patrick Steptoe reported the birth of the first test-tube baby in England [3]. Their innovative IVF method successfully treated a patient with damaged fallopian tubes, representing a significant advancement in reproductive medicine [4-7].

Dr. Mukherjee’s IVF techniques were notably advanced and meticulously documented. He addressed low sperm counts using gonadotropins, a method that would later become standard treatment. In a report dated October 19, 1978, submitted to the Director of Health Services, Government of West Bengal, Mukherjee outlined his ovarian stimulation protocol with human menopausal gonadotropin and human chorionic gonadotropin (hCG), a strategy that was ahead of its time compared to similar methods adopted later by others.

For oocyte retrieval, Mukherjee introduced a pioneering transvaginal technique via colpotomy, which was simpler and more direct than the laparoscopy used by British teams. This method involved accessing the ovaries through a small incision in the vaginal wall, reflecting his innovative approach.

Mukherjee’s in vitro culture techniques included incubating oocytes with processed semen and using a cervical-uterine fluid mixture for embryo development, aligning closely with current standards. His groundbreaking work in cryopreservation involved gradually freezing embryos with dimethyl sulfoxide and successfully thawing them for implantation, leading to the birth of a healthy baby.

Remarkably, Mukherjee’s cryopreservation techniques, including the successful storage and thawing of eight-cell embryos, were reported in 1978 - five years before similar successes were achieved elsewhere. His early accomplishments in this field highlighted his pioneering contributions to reproductive technology long before they were widely recognized.

Criticism after his landmark breakthrough

In 1978, the Government of West Bengal established an inquiry committee under the Indian Medical Council (West Bengal Chapter) and the West Bengal Obstetrics and Gynecology Association to evaluate Dr. Mukherjee’s IVF research. Despite submitting a comprehensive report, supported by two other scientists, Mukherjee’s claims were dismissed by the committee, which lacked expertise in human reproduction. The committee deemed his work “unbelievable” and “absurd,” ultimately labeling it as “bogus.” Mukherjee also faced public scorn and professional humiliation in Calcutta, and his findings were briefly published in the Indian Journal of Cryogenics in 1979 [4].

In 1980, Mukherjee was deliberately transferred to R.G. Kar Medical College in Calcutta, far from his home. The following year, he was reassigned to the Regional Institute of Ophthalmology at Medical College, Calcutta. These relocations severely hampered his ability to continue his research in reproductive medicine. Overwhelmed by mental pressure, insults, and defamation, he took his own life on June 18, 1981. In his final note, he reportedly wrote, “I can’t wait every day for a heart attack to kill me” [2].

Delayed acknowledgment of Dr. Mukherjee’s contributions

In 1986, Dr. T. C. Anand Kumar and Dr. Indira Hinduja reported the birth of India’s first “scientifically” documented IVF baby, Harsha, at the Indian Council of Medical Research’s (ICMR) Institute for Research in Reproduction in collaboration with KEM Hospital Mumbai [8]. This milestone was featured in the ICMR Bulletin and the Journal of In Vitro Fertilization and Embryo Transfer.

Although Dr. Anand Kumar could not attend the Dr. Subhas Mukherjee Memorial Oration in 1983, he later engaged with Mukherjee’s work during the 1997 Dr. Subhas Mukherjee Memorial Lecture. Anand Kumar acknowledged Mukherjee’s pioneering efforts, emphasizing that “Durga” was India’s first IVF baby, and compiled Mukherjee’s contributions in a 1997 Current Science article titled “Architect of India’s First Test Tube Baby: Dr. Subhas Mukherjee” [9].

Dr. Anand Kumar praised Mukherjee for his advanced ovarian stimulation techniques and credited his innovations in IVF and embryo cryopreservation as foundational to modern assisted reproduction practices. In 2002, the ICMR officially recognized Mukherjee’s contributions, largely due to Anand Kumar’s efforts.

Other notable contributions to the field of reproductive medicine

In addition to his groundbreaking work in IVF, Dr. Mukherjee made several other significant contributions to reproductive medicine. His research spanned various aspects of reproductive health and endocrinology, showcasing his broad expertise and innovative approach [10].

He expanded on Theodore Langham’s 1870 research by demonstrating that hCG-like substances from nonpregnant endometrial tissue are crucial for menstrual health and corpus luteum maintenance. In 1973, Dr. Mukherjee investigated Testicular Feminization Syndrome, delving into aspects of transsexuality. He also conducted pioneering studies on the effects of testosterone on reproductive tissues in rats, which advanced treatment for female infertility. Additionally, he proposed a link between emotional stress and polycystic ovarian disorder, presenting his findings at the 1977 International Congress on Physiological Science in Paris.

Posthumous accolades

Following his death, Dr. Mukherjee was posthumously honored for his remarkable contributions to reproductive medicine [2]. In 1982, the Indian Cryogenics Council established the “Dr. Subhas Mukherjee Memorial Oration” to commemorate his work. Three years later, in 1985, they, together with Behala Balananda Brahmachari Hospital and Jadavpur University, inaugurated the Dr. Subhas Mukherjee Memorial Reproductive Biology Research Centre in Behala, Calcutta, with Prof. Ashok Mitre, IAS, presiding over the event.

In 1994, a new division of the Dr. Subhas Mukherjee Memorial Reproductive Biology Research Centre was inaugurated by Mr. Abdur Razzak Molla, Hon'ble West Bengal State Minister for Food Processing Industries. The ICMR officially recognized Dr. Mukherjee’s pioneering work in 2002, including it in their “National Guidelines for Accreditation, Supervision, and Regulation of ART Clinics in India.”

In 2007, Dr. Mukherjee was included in the “Dictionary of Medical Biography” published by Greenwood Press, alongside notable Calcutta-based doctors Ronald Ross and U. N. Brahmachari. That same year, the Brazilian Medical Society celebrated his contributions and presented mementos to both the Dr. Subhas Mukherjee Memorial Reproductive Biology Research Centre and Dr. T. C. Anand Kumar.

In 2011, Dr. Mukherjee’s “Life and Work” was featured in the literature journal DAWN. He was also posthumously awarded an Honorary DSc degree by then Central Finance Minister Sri Pranab Mukherjee at the Indian Statistical Institute, Baranagar, Kolkata. In 2012, the ICMR announced the Dr. Subhas Mukherjee Award in his memory.

In 2013, Dr. Mukherjee was commemorated in a bulletin for the 55th Foundation Day of the Institute of Post Graduate Medical Education and Research. He was further acknowledged in a seminar organized by the Indian Society for Assisted Reproduction and Bengal Obstetrics and Gynecology Society, where the first Dr. Subhas Mukherjee Memorial Lecture was held, featuring a paper on “ART-Past, Present, and Future.”

In 2020, the ICMR-National Institute for Research in Reproductive Health, Mumbai, published a book titled “Dr. Subhas Mukherjee: A Visionary and Pioneer of IVF” as part of the Golden Jubilee celebrations of the institute. This initiative, supported by the Indian National Science Academy, aimed to raise awareness about Dr. Mukherjee’s achievements.

## Conclusions

Dr. Subhas Mukherjee’s pioneering work in ART, especially IVF, was unfortunately not recognized during his lifetime despite his groundbreaking achievements, including the development of advanced ovarian stimulation methods and embryo cryopreservation. His major milestones, such as the successful birth of India’s first test-tube baby, “Durga,” were met with skepticism and professional difficulties, leading to a lack of acknowledgment and considerable personal hardship.

It was only after his death that Dr. Mukherjee’s contributions began to receive the recognition they deserved, largely due to the efforts of Dr. T. C. Anand Kumar. Today, Dr. Mukherjee’s legacy is celebrated for his significant role in shaping modern assisted reproductive technologies, solidifying his esteemed position in the history of medical science.
